# An acoustic detection dataset of birds (Aves) in montane forests using a deep learning approach

**DOI:** 10.3897/BDJ.11.e97811

**Published:** 2023-02-24

**Authors:** Shih-Hung Wu, Jerome Chie-Jen Ko, Ruey-Shing Lin, Wen-Ling Tsai, Hsueh-Wen Chang

**Affiliations:** 1 Department of Biological Sciences, National Sun Yat-Sen University, Kaohsiung, Taiwan Department of Biological Sciences, National Sun Yat-Sen University Kaohsiung Taiwan; 2 Endemic Species Research Institute, Nantou, Taiwan Endemic Species Research Institute Nantou Taiwan; 3 Institute of Ecology and Evolutionary Biology, National Taiwan University, Taipei, Taiwan Institute of Ecology and Evolutionary Biology, National Taiwan University Taipei Taiwan; 4 Yushan National Park Headquarters, Nantou, Taiwan Yushan National Park Headquarters Nantou Taiwan

**Keywords:** passive acoustic monitoring, Yushan National Park, Aves, SILIC, automated sound identification, biodiversity, soundscape

## Abstract

**Background:**

Long-term monitoring is needed to understand the statuses and trends of wildlife communities in montane forests, such as those in Yushan National Park (YSNP), Taiwan. Integrating passive acoustic monitoring (PAM) with an automated sound identifier, a long-term biodiversity monitoring project containing six PAM stations, was launched in YSNP in January 2020 and is currently ongoing. SILIC, an automated wildlife sound identification model, was used to extract sounds and species information from the recordings collected. Animal vocal activity can reflect their breeding status, behaviour, population, movement and distribution, which may be affected by factors, such as habitat loss, climate change and human activity. This massive amount of wildlife vocalisation dataset can provide essential information for the National Park's headquarters on resource management and decision-making. It can also be valuable for those studying the effects of climate change on animal distribution and behaviour at a regional or global scale.

**New information:**

To our best knowledge, this is the first open-access dataset with species occurrence data extracted from sounds in soundscape recordings by artificial intelligence. We obtained seven bird species for the first release, with more bird species and other taxa, such as mammals and frogs, to be updated annually. Raw recordings containing over 1.7 million one-minute recordings collected between the years 2020 and 2021 were analysed and SILIC identified 6,243,820 vocalisations of seven bird species in 439,275 recordings. The automatic detection had a precision of 0.95 and the recall ranged from 0.48 to 0.80. In terms of the balance between precision and recall, we prioritised increasing precision over recall in order to minimise false positive detections. In this dataset, we summarised the count of vocalisations detected per sound class per recording which resulted in 802,670 occurrence records. Unlike data from traditional human observation methods, the number of observations in the Darwin Core "organismQuantity" column refers to the number of vocalisations detected for a specific bird species and cannot be directly linked to the number of individuals.

We expect our dataset will be able to help fill the data gaps of fine-scale avian temporal activity patterns in montane forests and contribute to studies concerning the impacts of climate change on montane forest ecosystems on regional or global scales.

## Introduction

Montane forests are biodiversity hotspots with diverse species richness and compositions along an altitudinal gradient (Körner 2004, Richter 2008, Willig and Presley 2015). However, they are vulnerable to climate change that may impact biodiversity and reshape species distributions (Foster 2001, Beniston 2003, Antonelli et al. 2018). Long-term monitoring is needed to understand the statuses and trends of wildlife communities in a montane forest. For such purposes, birds are commonly used as indicators for biodiversity and climate change (Schulze et al. 2004, Butchart et al. 2010, Fraixedas et al. 2020, Oettel and Lapin 2021). However, monitoring montane birds is challenging because of economic issues and the inaccessibility of locations (Chamberlain et al. 2011, Şekercioğlu et al. 2012). With limited resources, community-based citizen science programmes such as the UK Breeding Bird Survey and eBird help to acquire data at large temporal and spatial scales, critical to long-term monitoring (Horns et al. 2018, Martay et al. 2018). However, the training of volunteers and the validation of data should be applied carefully to minimise the biases in locations, preferred taxa and variation in sampling effort and observer skill (Dickinson et al. 2010, Kosmala et al. 2016). Instead, a regular, cost-effective, systematic and automatic monitoring method that can be conducted for a long period may help gather data on large scales with stable quality.

Passive acoustic monitoring is gaining ground in ecology because it utilises autonomous recording units (ARUs) that can be deployed in a variety of environments for long periods of time, allowing for the collection of large amounts of high-resolution soundscape data for biodiversity monitoring (Gibb et al. 2018, Zwerts et al. 2021). The advantages of no observer bias, few skilled experts needed and low maintenance cost promote PAM to be a highly cost-effective method in long-term monitoring, particularly for birds (Sugai et al. 2018, Darras et al. 2019). Its feasibility has been proven in investigating montane bird communities (Campos-Cerqueira et al. 2017). However, manually extracting species and quantity information from a large number of recordings is time-consuming and labour-intensive. Fortunately, machine-learning-based automatic sound identification tools, such as BirdNET (Kahl et al. 2021) and SILIC (Wu et al. 2022) have been developed to overcome these problems.

To monitor the montane forest biodiversity in Yushan National Park (YSNP), we initiated a passive acoustic monitoring project and deployed six PAM stations as a start in 2020. Our goal was to use animal vocal activity as an indicator to assess the status and trends of animal populations. This dataset is our first result and contains 6,243,820 vocalisations of seven montane forest bird species recorded in 2020 and 2021. These vocalisations were automatically identified from 1,776,492 one-minute recordings (~ 29,608 hours) using SILIC. The species, temporal and spatial coverages will be updated annually.

In most traditional human observation methods for bird monitoring, an occurrence means the existence of one or more organisms at a specific place and time. However, in this dataset, the subjects are vocalisations, not organisms, because we cannot identify the individuals who produced the vocalisations in the recordings. Thus, we treated the number of vocalisations detected for each sound class in a specific recording as an occurrence. This means that the number of observations in the "organismQuantity" column refers to the number of vocalisations detected for a specific bird species and cannot be directly inferred as the number of individuals, although some studies have found a positive relationship between the two (Sebastián-González et al. 2018, Pérez-Granados et al. 2019).

Animal vocal activity can provide valuable insights into their behaviour, population trends, migration phenology and changes in distribution, which may be influenced by habitat loss, climate change and human activity (Shonfield and Bayne 2017, Teixeira et al. 2019, Lewis et al. 2020, Pérez‐Granados and Traba 2021). This dataset can be of great value not only for our management and decision-making, but also for researchers studying the effects of human activity and climate change on animal ecology at a regional or global scale. However, it should be noted that the six PAM stations, each containing only one ARU, may not fully represent the animal population in similar habitats or at similar altitudes. Additionally, the detection range of the ARUs is unknown so we could not evaluate the volume of space sampled. Nor do we know the volume of and its effect on the automatic detection process. Therefore, we recommend analysing these data on a temporal scale and focusing on species presence rather than abundance. Additionally, by sub-sampling this dataset and reviewing the original audio recordings manually, users could create a large ground-truth dataset, which could be used to develop and evaluate new sound identification models.

## Project description

### Title

Passive acoustic monitoring at Yushan National Park

### Personnel

The PAM stations were maintained by the YSNP Headquarters and the data were archived, managed, analysed and prepared for release by the Endemic Species Research Institute (ESRI), Taiwan.

## Sampling methods

### Quality control

The functionality of the ARUs was checked on a monthly basis. The SILIC detector was used to detect sound labels of target sound classes and produced information containing the filename, sound class ID, start and end time, low and high frequency and a confidence score. To evaluate the performance of SILIC on our soundscape recordings, we randomly selected 150 labels for each sound class and reviewed them manually to create a ground-truth dataset. The predicted results of SILIC were then compared with the ground-truth to produce a confusion matrix that includes four parameters: true positive (TP), true negative (TN), false positive (FP) and false negative (FN). The precision (TP/(TP+FP)), recall (TP/(TP+FN)) and accuracy ((TP+TN)/(TP+FP+TN+FN)) were also calculated. When increasing the confidence score, precision increases, but recall decreases. To minimise false positive detections in the released dataset, we prioritised increasing precision over recall. Additionally, we chose to use precision instead of accuracy as a measure to prevent bias due to the large number of true negative detections that are not included in the released dataset. Finally, we selected the minimal confidence threshold necessary to achieve a precision of 0.95 or higher for each sound class. To further evaluate the performance of SILIC, we also calculated additional metrics, such as the area under the receiver operating characteristic curve (AUC) and the area underneath the precision-recall curve (AP or average precision). The sound class, confidence threshold and performance metrics are shown in Table 1 and the precision and recall curves for each sound class can be found in Suppl. material 1. The equations of performance metrics are explained in Suppl. material 3.

### Step description

1. In this project, one Song Meter SM4 or Song Meter Mini made by Wildlife Acoustic Inc. was deployed at each PAM station as the autonomous recording unit (ARU). The ARUs were mounted on trees approximately 1.5 metres above the ground and shielded by sound-absorbing canopies to reduce the impact of raindrop noise and ensure that the microphone windscreens remained dry. This is because a wet windscreen can impede the transmission of sound (The photos of PAM stations are shown in Suppl. material 2). Due to the resources required for power supply, data storage and acoustic analysis of continuous recording for a long-term monitoring project, all ARUs were configured to record one-min recordings every three minutes in stereo, 16-bit WAV format at a sampling rate of 44.1 kHz.
2. Memory cards storing acoustic data were replaced monthly and two copies of files were archived separately in local storages and Google Drive for data safety.
3. The “exp24” model in SILIC (https://github.com/RedbirdTaiwan/silic/blob/master/model/exp24) was utilised to automatically detect animal vocalisations in the recordings. Following the detection process outlined in Wu et al. (2022), each one-minute recording was transformed into a set of 3-second spectrogram clips and detected using a 1-second sliding window. The detection process produced sound labels containing the information of filename, sound class ID, start/end time and low/high frequency (i.e. a bounding box in the time and frequency domains) and confidence score of each detected sound label, as one sound object might be identified multiple times when applying a sliding window with an overlap, especially for those with duration longer than 3 seconds. For bounding boxes with the same sound class, if either the intersection area of two overlapping bounding boxes divided by the area of the smaller box was greater than 0.5 or the intersection area divided by the union area was greater than 0.25, the two bounding boxes were merged.
4. One hundred and fifty (150) random labels of each sound class were sampled to evaluate the performance metrics including the precision, recall, AUC and AP (the equations are available in Suppl. material 3).
5. To minimise false positive detections in the released dataset, the confidence threshold for each sound class was chosen when the precision reached 0.95. All labels of each sound class with a confidence score above the threshold were considered as positive detections.
6. In this dataset, one recording is treated as one sampling event. To reduce storage requirements, we summarised the positive detections in the same recordings (events) by counting the number of vocalisations of each species as the number of observations and filled in the column "organismQuantity". It is important to note that the number of observations in the dataset does not represent the number of individual organisms as we cannot identify the individuals who produced the sounds in the recordings.

## Geographic coverage

### Description

The study area was located in the southern area of YSNP, a typical montane ecosystem in central Taiwan. Six PAM stations were deployed between Meishan and Yako along the Southern Cross-Island Highway, with an elevation range from 1,264 m above sea level (MSC01) to 2,739 m (WK01). The longest distance between any two stations was around 11.4 km and the shortest distance was 500 m. The habitat types vary from lower (1,264 m) to higher (2,739 m) elevation, including sub-montane evergreen broad-leaved forests (C2A07), montane evergreen broad-leaved cloud forests (C2A05), montane mixed cloud forests (C2A03) and upper-montane coniferous forests (C1A02) (Li et al. 2013, Fig. 1, Table 2).

### Coordinates

23.257 and 23.288 Latitude; 120.826 and 120.955 Longitude.

## Taxonomic coverage

### Description

The taxonomic coverage will increase with the version and precision of SILIC, which is used to detect animal vocalisations automatically in soundscape recordings. As SILIC supports multiple sound classes for a single species, we selected one normal sound class for each species. In version 1.5, we selected seven bird species as pioneers, including the White-eared Sibia *Heterophasiaauricularis* (WS), Taiwan Barbet *Psilopogonnuchalis* (TB), Steere's Liocichla *Liocichlasteerii* (SL), Taiwan Yuhina *Yuhinabrunneiceps* (TY), Gray-chinned Minivet *Pericrocotussolaris* (GM), White-tailed Robin *Myiomelaleucura* (WR) and Large-billed Crow *Corvusmacrorhynchos* (LC) (Table 3). For species with multiple sound classes available in SILIC, we selected the most frequently heard sound type.

### Taxa included

**Table:** 

Rank	Scientific Name	Common Name
species	*Heterophasiaauricularis*	White-eared Sibia
species	*Psilopogonnuchalis*	Taiwan Barbet
species	*Liocichlasteerii*	Steere's Liocichla
species	*Yuhinabrunneiceps*	Taiwan Yuhina
species	*Pericrocotussolaris*	Gray-chinned Minivet
species	*Myiomelaleucura*	White-tailed Robin
species	*Corvusmacrorhynchos*	Large-billed Crow

## Temporal coverage

**Data range:** 2020-1-20 – 2021-12-31.

### Notes

One PAM station was deployed on 20 January 2020, four on 21 January 2020 and one on 22 January 2020. The latest date of the recordings analysed in this dataset was 31 December 2021.

## Usage licence

### Usage licence

Other

### IP rights notes

Creative Commons Attribution (CC-BY) 4.0 License

## Data resources

### Data package title

Darwin Core Archive Acoustic detections of birds using SILIC in Yushan National Park, Taiwan

### Resource link

https://ipt.taibif.tw/archive.do?r=silic-ysnp

### Alternative identifiers

https://ipt.taibif.tw/resource?r=silic-ysnp

### Number of data sets

1

### Data set 1.

#### Data set name

Acoustic detections of birds using the SILIC in Yushan National Park, Taiwan

#### Data format

Darwin Core Archive format

#### Character set

UTF-8

#### Download URL

https://ipt.taibif.tw/archive.do?r=silic-ysnp

#### Data format version

1.0

#### Description

**Figure 2.**
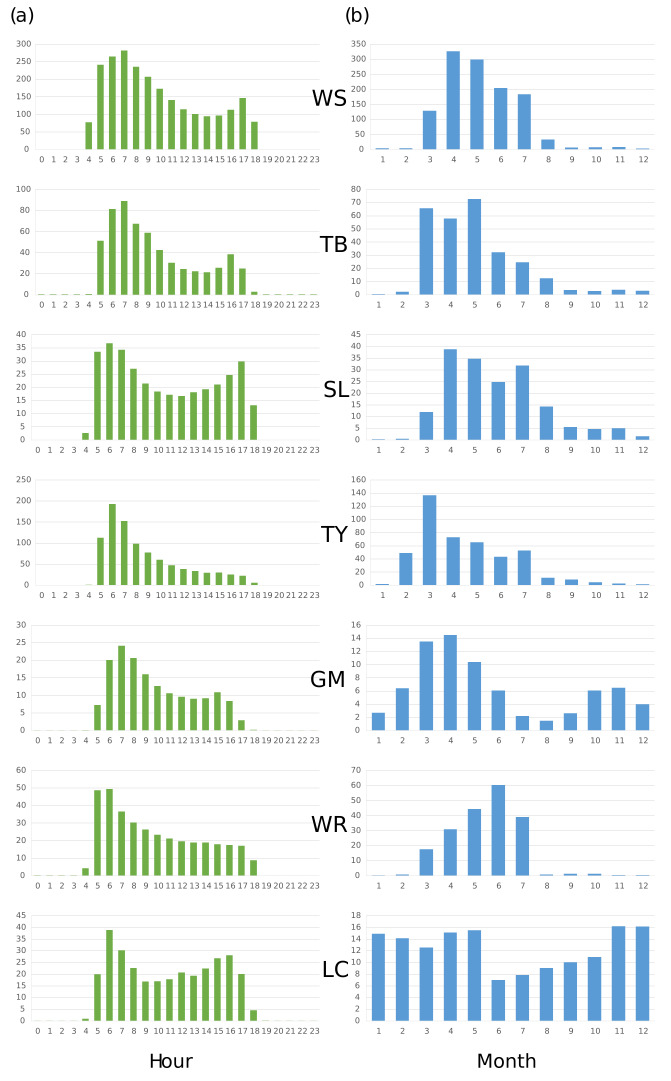
The diurnal (**a**) and seasonal (**b**) patterns of the vocal activities of White-eared Sibia (WS), Taiwan Barbet (TB), Steere's Liocichla (SL), Taiwan Yuhina (TY), Gray-chinned Minivet (GM), White-tailed Robin (WR) and Large-billed Crow (LC) provide important biological information for biodiversity studies and management. The Y-axis is the mean number of vocalisations per hour and the X-axis is hour for diurnal pattern and month for seasonal one.

The dataset describes 439,275 one-minute recording events, with 6,243,820 vocalisations of seven bird species identified and summarised into 802,670 occurrence records (Tables 4, 5). The original 1,776,492 recordings are available on an online research data repository - depositar (https://pid.depositar.io/ark:37281/k5x86156b). With a time span of two full years and high temporal-resolution data (one recording per three minutes per day), we were able to identify clear daily and seasonal patterns of bird vocal activity (Fig. 2). The daily pattern with a highest peak in the morning, as well as the seasonal pattern peaking during the breeding season, are similar to those observed in other songbirds (Puswal et al. 2022). However, the seasonal pattern of the Large-billed Crow (LC) deviates from this trend as we used its call, rather than its song, as the target sound type. In addition, the Gray-chinned Minivet (GM) shows a small peak during the non-breeding period, which may correspond to the flocking behaviour observed (Kwok 2017).

**Data set 1.** 

Column label	Column description
eventID	An identifier for an Event.
samplingProtocol	The methods used during an Event.
sampleSizeValue	A numeric value for a time duration of a recording sample in an event.
sampleSizeUnit	The unit of the time duration.
eventDate	The date which an Event occurred.
eventTime	The time which an Event occurred.
eventRemarks	Notes about recording setups.
locationID	An identifier for locations.
decimalLatitude	The geographic latitude in decimal degrees.
decimalLongitude	The geographic longitude in decimal degrees.
geodeticDatum	The spatial reference system (SRS) of coordinates.
coordinateUncertaintyInMeters	The maximum acoustic detection range.
coordinatePrecision	A decimal representation of the precision of the coordinates.
type	The nature of the resource.
modified	Date on which the resource was changed.
basisOfRecord	The specific nature of the data record.
occurrenceID	An identifier for the Occurrence.
recordedBy	The names of people responsible for recording the original Occurrence.
organismQuantity	The quantity of vocalisations detected for a specific animal species within a 1-minute recording.
organismQuantityType	"Detected vocalisations" for a specific animal species. The detected vocalisations in this dataset were identified using the process described in the "Sampling methods" section, which employs the SILIC detector. It is important to note that not all vocalisations were detected and a small proportion may have been misidentified. Therefore, to ensure the reliability of our data, we aimed to maintain a precision rate of 0.95 for each sound class.
occurrenceStatus	A statement about the presence or absence of a Taxon at a Location.
associatedMedia	A URL of an audio file associated with the Occurrence.
occurrenceRemarks	The sound class id of SILIC exp 24 associated with the Occurrence.
scientificName	The full scientific name.
family	The full scientific name of the family.
taxonRank	The taxonomic rank of the scientificName.
vernacularName	A common name in Traditional Chinese.

## Supplementary Material

CB780832-9BEC-5834-841E-FF98CAD93808 10.3897/BDJ.11.e97811.suppl1 Supplementary material 1 The precision and recall curves of the seven target species / sound classes Data type images Brief description The precision (blue), recall (green) and F1-score (black) curves of (**a**) White-eared Sibia *Heterophasiaauricularis*, (**b**) Taiwan Barbet *Psilopogonnuchalis*, (**c**) Steere's Liocichla *Liocichlasteerii*, (**d**) Taiwan Yuhina *Yuhinabrunneiceps*, (**e**) Gray-chinned Minivet *Pericrocotussolaris*, (**f**) White-tailed Robin *Myiomelaleucura* and (**g**) Large-billed Crow *Corvusmacrorhynchos*; the red dash line showed the score of the threshold when the precision = 0.95. File: oo_769678.pdf https://binary.pensoft.net/file/769678 Shih-Hung Wu, Jerome Chie-Jen Ko, Ruey-Shing Lin, Wen-Ling Tsai, Hsueh-Wen Chang

09E46A35-86B7-5CAE-AF3A-9828EF8F5F16 10.3897/BDJ.11.e97811.suppl2 Supplementary material 2 The six PAM stations Data type images Brief description The setup environments of six PAM stations. File: oo_769679.pdf https://binary.pensoft.net/file/769679 Shih-Hung Wu, Jerome Chie-Jen Ko, Ruey-Shing Lin, Wen-Ling Tsai, Hsueh-Wen Chang

2700E0BC-B877-5AF0-AE55-C1E82CBAC5E6 10.3897/BDJ.11.e97811.suppl3 Supplementary material 3 Performance metrics Data type equations Brief description For performance evaluation, we applied the trained model on a test dataset and obtained the predicted class of each data. The predicted results were compared with the ground-truth to obtain a confusion matrix that indicates four parameters as true positive (TP), true negative (TN), false positive (FP) and false negative (FN) (Fig. S1). Then, we can calculate the performance metrics as precision (Eq. 1), recall (Eq. 2) and F1 score (Eq. 3). File: oo_769680.pdf https://binary.pensoft.net/file/769680 Shih-Hung Wu, Jerome Chie-Jen Ko, Ruey-Shing Lin, Wen-Ling Tsai, Hsueh-Wen Chang

## Figures and Tables

**Figure 1.**
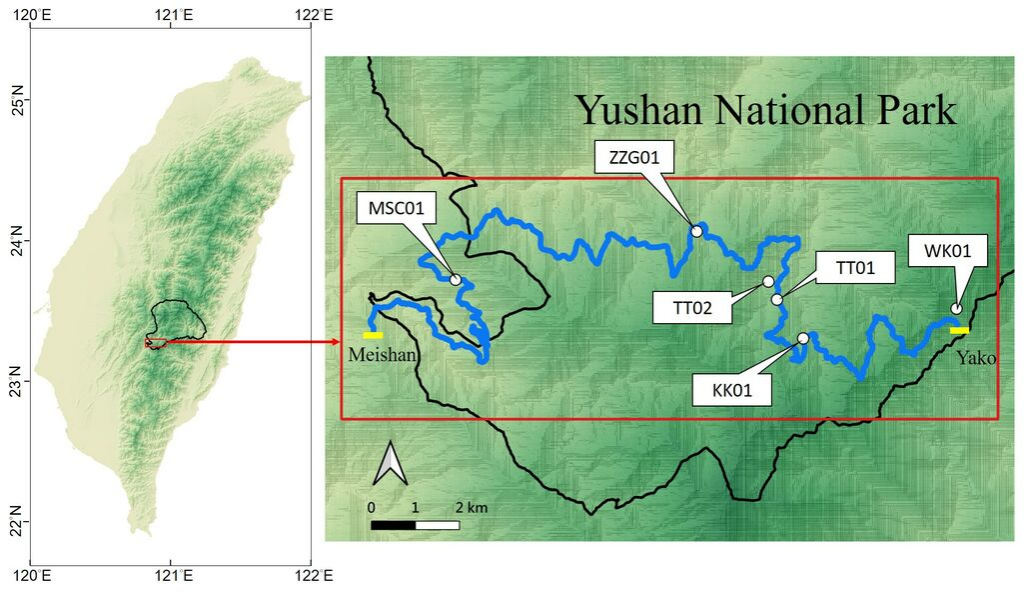
The study area located in the southern area (red rectangle) of YSNP (black line) in central Taiwan. Six PAM stations (white points) were deployed in the area between Meishan and Yako (yellow line) along the Southern Cross-Island Highway (blue line).

**Table 1.** The sound class, confidence threshold and performance metrics of seven target species.

Soundclass ID^##^	Species	Sound class^#^	Confidence threshold	Precision^##^	Recall^##^	AUC^##^	AP^##^
9	WS	S-01	0.54	0.95	0.53	0.90	0.94
28	TB	S-01	0.26	0.95	0.80	0.94	0.98
122	SL	S-01	0.73	0.95	0.48	0.91	0.91
324	TY	S-01	0.71	0.95	0.55	0.92	0.91
337	GM	U-01	0.57	0.95	0.72	0.94	0.95
361	WR	S-01	0.51	0.95	0.68	0.89	0.92
471	LC	C-01	0.48	0.95	0.64	0.90	0.96

# The sound-class IDs and classes were based on the sound-class list of the “exp24” model in SILIC (https://github.com/RedbirdTaiwan/silic/blob/master/model/exp24/soundclass.csv) for White-eared Sibia (WS), Taiwan Barbet (TB), Steere's Liocichla (SL), Taiwan Yuhina (TY), Gray-chinned Minivet (GM), White-tailed Robin (WR) and Large-billed Crow (LC).

## The equations of the performance metrics are shown in Suppl. material 3 and the precision and recall curves are shown in Suppl. material 1.

**Table 2.** The characters of the six PAM stations.

Site ID	Site name	Longitude (degree)	Latitude (degree)	Elevation (m a.s.l.)	Habitat type^#^
MSC01	Meishan	120.8440	23.2755	1,264	C2A07
ZZG01	Jhongjhihguan	120.8975	23.2862	2,047	C2A05
TT01	Tianchih (lower)	120.9153	23.2711	2,303	C2A05
TT02	Tianchih (upper)	120.9134	23.2751	2,366	C2A03
KK01	Kuaigu	120.9211	23.2625	2,429	C2A03
WK01	Yako	120.9551	23.2691	2,739	C1A02

# The habitat types followed the classification of Li et al. (2013) which were sub-montane evergreen broad-leaved forests (C2A07), montane evergreen broad-leaved cloud forests (C2A05), montane mixed cloud forests (C2A03) and upper-montane coniferous forests (C1A02).

**Table 3.** The acoustic attributes of the seven target species.

Soundclass ID^#^	Species	Sound class^#^	Mean min. frequency (Hz) ^#^	Mean max. frequency (Hz) ^#^	Mean duration (ms)
9	WS	S-01	1908	4390	827
28	TB	S-01	738	1273	429
122	SL	S-01	2661	5386	1045
324	TY	S-01	2044	5074	718
337	GM	U-01	4206	6837	451
361	WR	S-01	2928	4916	1026
471	LC	C-01	519	1666	275

# The sound-class IDs, classes and frequencies were based on the sound-class list of the “exp24” model in SILIC (https://github.com/RedbirdTaiwan/silic/blob/master/model/exp24/soundclass.csv) for White-eared Sibia (WS), Taiwan Barbet (TB), Steere's Liocichla (SL), Taiwan Yuhina (TY), Gray-chinned Minivet (GM), White-tailed Robin (WR) and Large-billed Crow (LC).

**Table 4.** The vocalisations of each PAM station for White-eared Sibia (WS), Taiwan Barbet (TB), Steere's Liocichla (SL), Taiwan Yuhina (TY), Gray-chinned Minivet (GM), White-tailed Robin (WR) and Large-billed Crow (LC).

Species	Vocalisations						Total
	MSC01	ZZG01	TT01	TT02	KK01	WK01	
WS	687,916	959,708	841,909	136,421	285,879	17,115	2,928,948
TB	585,618	118,193	11,087	2,770	2,154	5,699	725,521
SL	29,903	131,440	26,096	114,079	67,361	43,894	412,773
TY	149,708	108,098	259,848	116,172	329,806	185,680	1,149,312
GM	86,212	37,905	39,968	2,604	32,755	1,685	201,129
WR	32,108	57,846	221,177	49,512	80,610	4,847	446,100
LC	40,074	92,710	108,110	105,059	17,776	16,308	380,037
總計	1,611,539	1,505,900	1,508,195	526,617	816,341	275,228	6,243,820

**Table 5.** The occurrences of each PAM station for White-eared Sibia (WS), Taiwan Barbet (TB), Steere's Liocichla (SL), Taiwan Yuhina (TY), Gray-chinned Minivet (GM), White-tailed Robin (WR) and Large-billed Crow (LC).

Species	Occurrences						Total
	MSC01	ZZG01	TT01	TT02	KK01	WK01	
WS	56,320	62,284	54,294	26,063	35,765	9,400	244,126
TB	30,550	11,388	3,299	1,305	1,981	5,396	53,919
SL	9,293	25,891	9,432	20,320	19,351	10,813	95,100
TY	25,082	25,672	36,792	19,485	36,090	24,329	167,450
GM	14,604	7,972	7,375	1,268	6,062	1,421	38,702
WR	13,708	20,546	41,174	18,627	21,389	3,371	118,815
LC	7,883	15,204	24,934	25,515	5,943	5,079	84,558
總計	157,440	168,957	177,300	112,583	126,581	59,809	802,670
