# Supplementary material for: An acoustic detection dataset of birds (Aves) in montane forests using a deep learning approach
Source: Biodivers Data J. 2023 Feb 24;11:e97811. doi: 10.3897/BDJ.11.e97811 (PMC10848598; doi:10.3897/BDJ.11.e97811)

The precision and recall curves of the seven target species / sound classes

The precision (blue), recall (green), and F1-score (black) curves of (a) White-eared Sibia *Heterophasia auricularis*, (b) Taiwan Barbet *Psilopogon nuchalis*, (c) Steere's Liocichla *Liocichla steerii*, (d) Taiwan Yuhina *Yuhina brunneiceps*, (e) Gray-chinned Minivet *Pericrocotus solaris*, (f) White-tailed Robin *Myiomela leucura*, and (g) Large-billed Crow *Corvus macrorhynchos*, the red dash line showed the score of the threshold when the precision = 0.95.

(a) White-eared Sibia *Heterophasia auricularis*

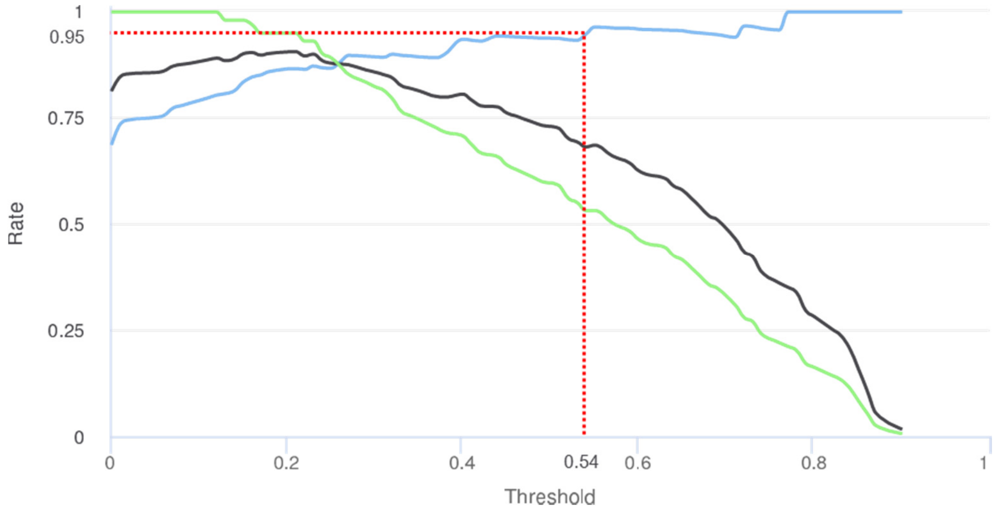

(b) Taiwan Barbet *Psilopogon nuchalis*

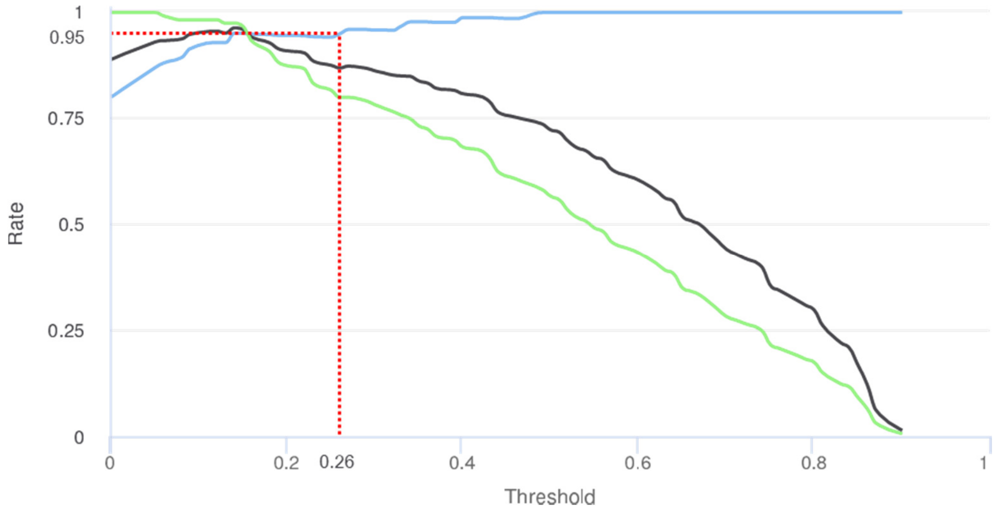

(c) Steere's Liocichla *Liocichla steerii*,

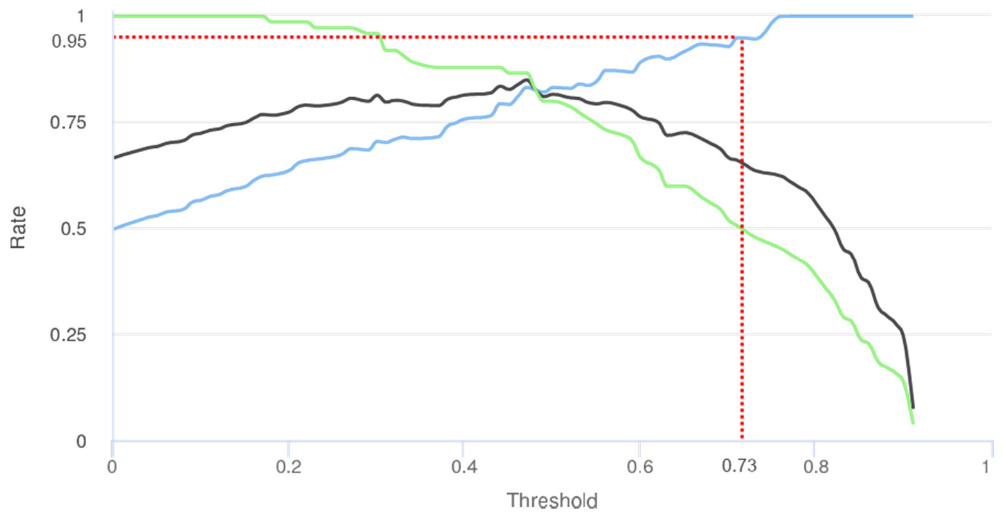

(d) Taiwan Yuhina *Yuhina brunneiceps*

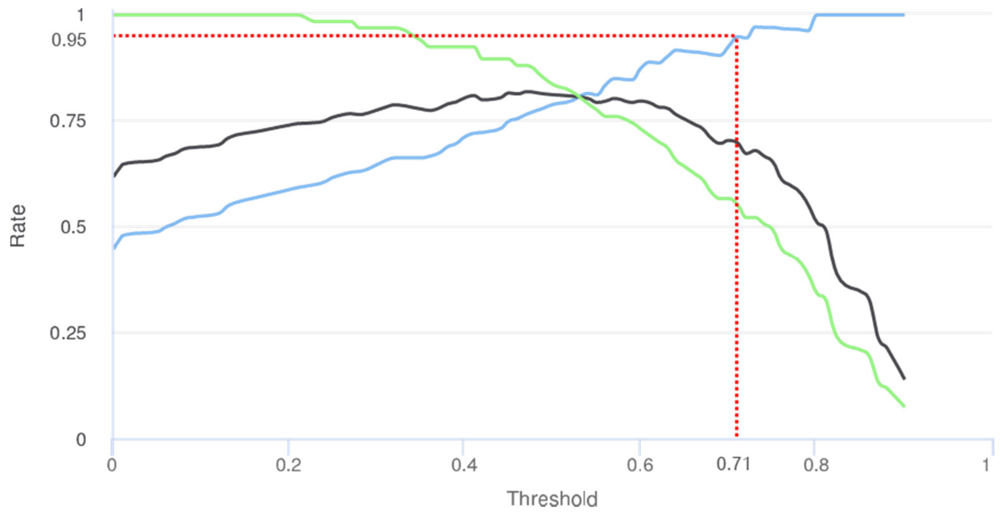

(e) Gray-chinned Minivet *Pericrocotus solaris*

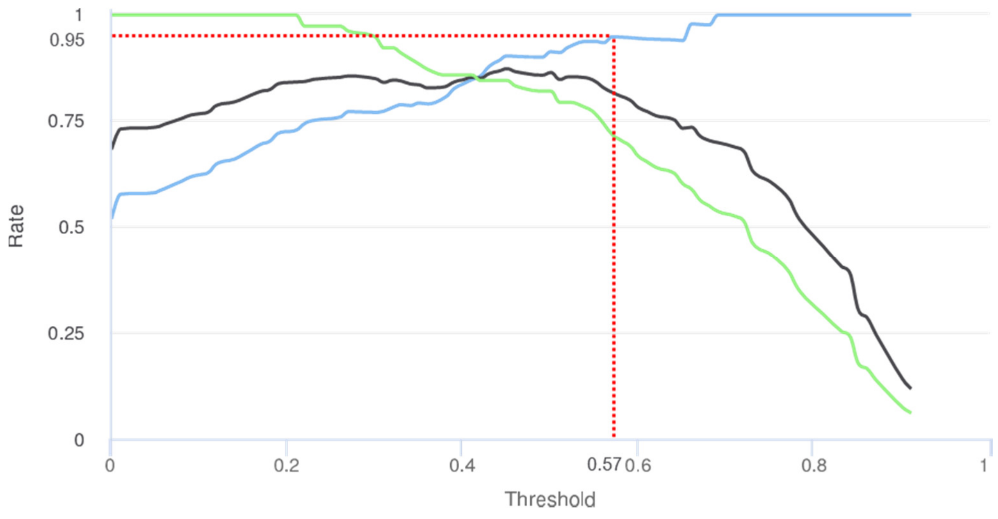

(f) White-tailed Robin *Myiomela leucura*

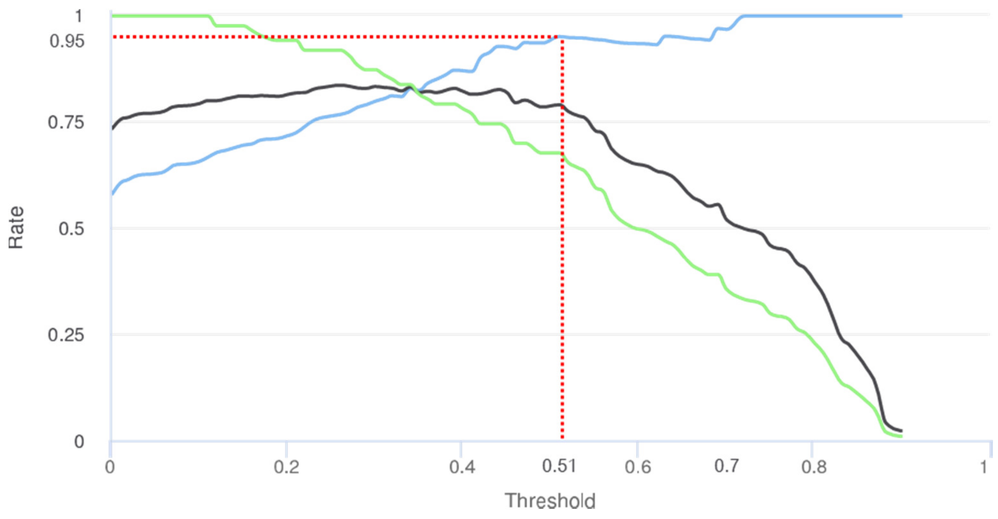

(g) Large-billed Crow *Corvus macrorhynchos*

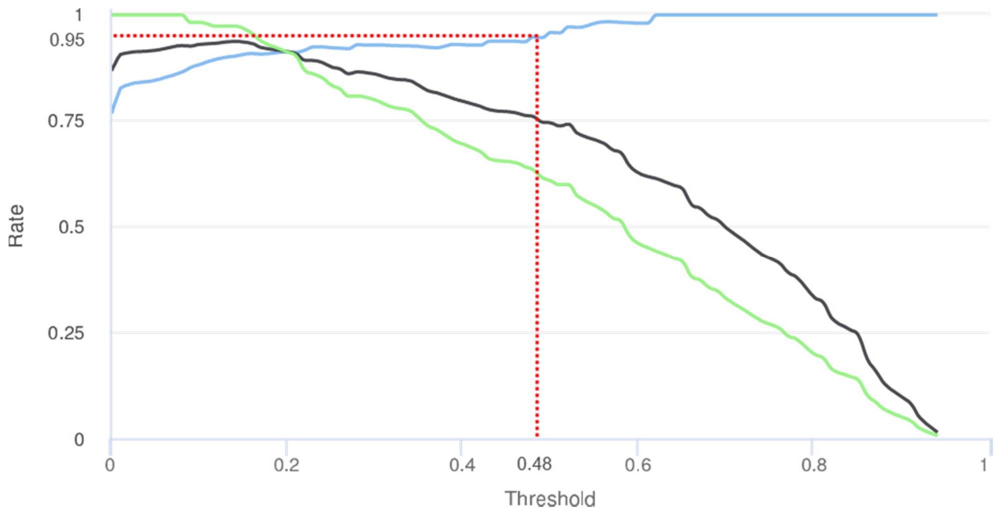

Supplement: Supplementary material 1 — The precision and recall curves of the seven target species / sound classes [file bdj-11-e97811-s001.pdf]
