# Supplementary material for: An acoustic detection dataset of birds (Aves) in montane forests using a deep learning approach
Source: Biodivers Data J. 2023 Feb 24;11:e97811. doi: 10.3897/BDJ.11.e97811 (PMC10848598; doi:10.3897/BDJ.11.e97811)
Supplement: Supplementary material 2 — The six PAM stations [file bdj-11-e97811-s002.pdf]

The six PAM stations

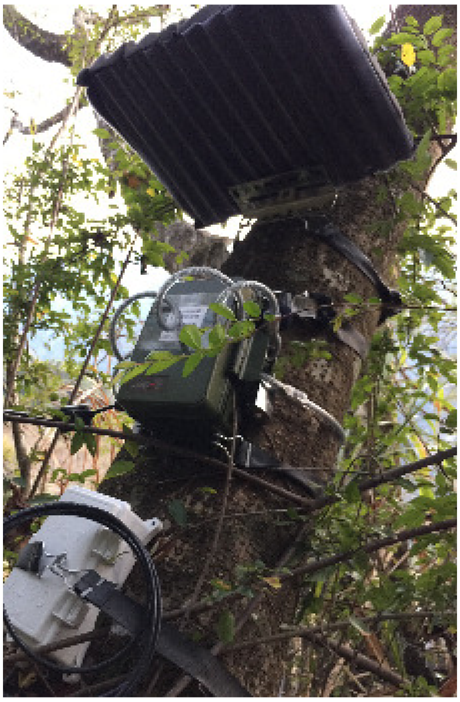

Meishan (MSC01)

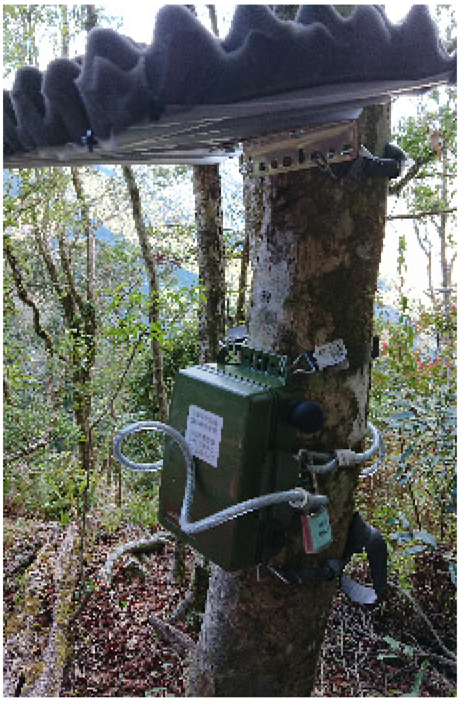

Jhongjhihguan (ZZG01)

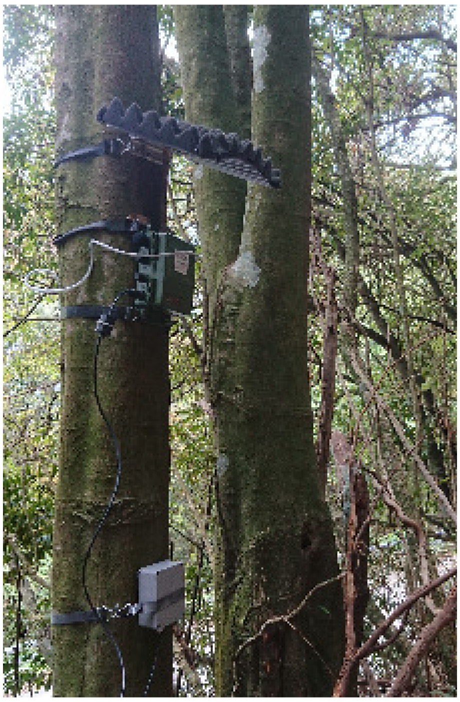

Tianchih(lower) (TT01)

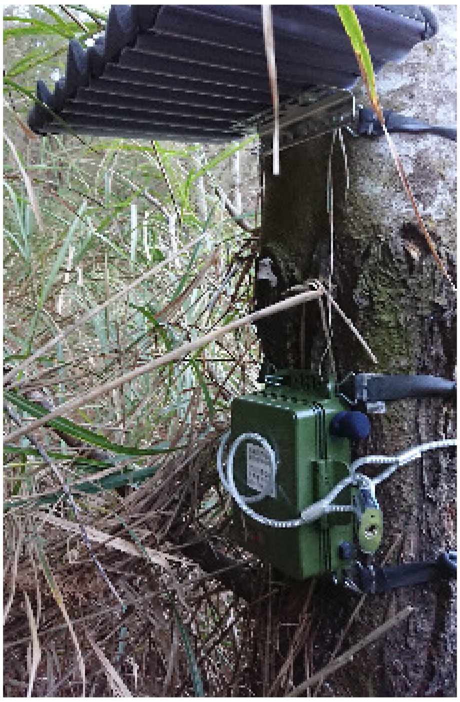

Tianchih(upper) (TT02)

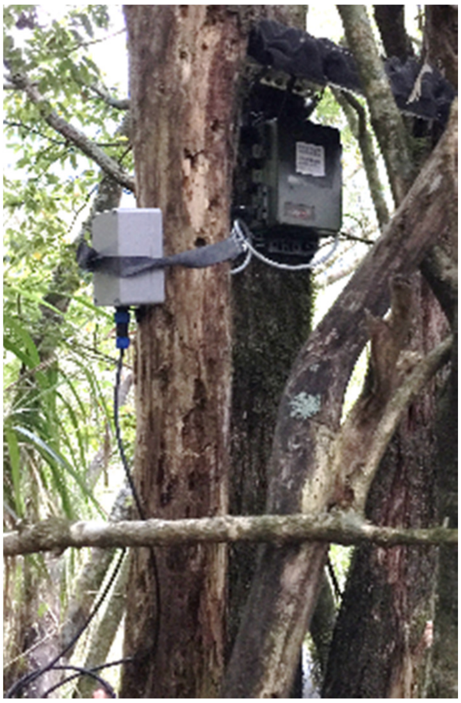

Kuaigu (KK01)

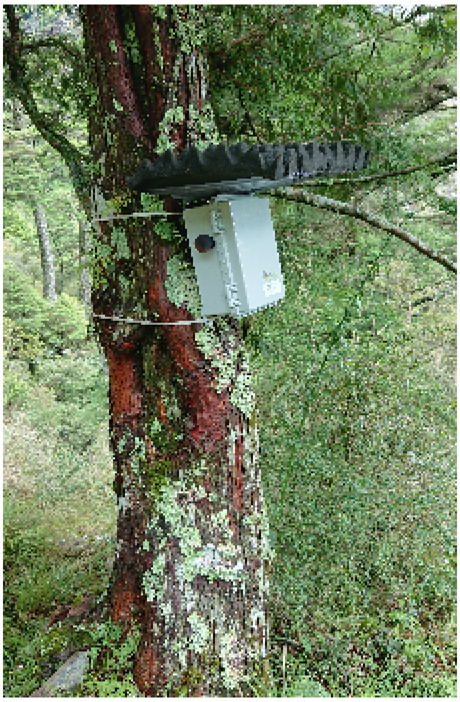

Yako (WK01)
