# Supplementary material for: An acoustic detection dataset of birds (Aves) in montane forests using a deep learning approach
Source: Biodivers Data J. 2023 Feb 24;11:e97811. doi: 10.3897/BDJ.11.e97811 (PMC10848598; doi:10.3897/BDJ.11.e97811)
Supplement: Supplementary material 3 — Performance metrics [file bdj-11-e97811-s003.pdf]

Performance metrics

For performance evaluation, we applied the trained model on an unseen test dataset and got the predicted class of each data. The predicted results were compared with the ground truth to get a confusion matrix that indicates four parameters as true positive (TP), true negative (TN), false positive (FP) and false negative (FN) (Fig. S1). Then, we can calculate the performance metrics as precision (Eq. 1), recall (Eq. 2) and F1 score (Eq. 3).

1. $$\text{Precision} = \frac{\text{TP}}{(\text{TP+FP})}$$

2. $$\text{Recall} = \frac{\text{TP}}{(\text{TP+FN})}$$

3. $$\text{F1 score} = \frac{2*(\text{Recall}*\text{Precision})}{(\text{Recall+Precision})}$$

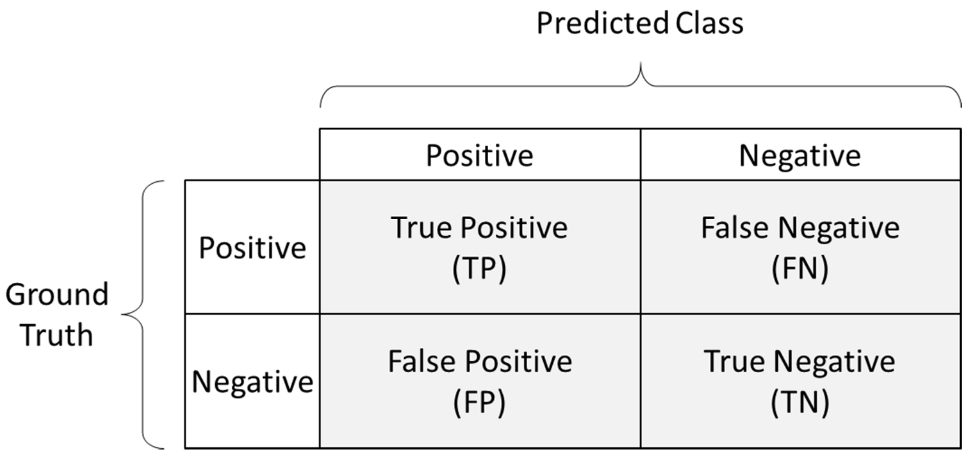

Figure S1.
Confusion matrix

Precision-recall curve (PR curve), average precision (AP) and mean AP (mAP)
For each class, when different threshold score is selected, different values of parameters will be calculated. A PR curve (precision-recall curve) is so plotted with precision (y-axis) and recall (x-axis) along with the changes in threshold score. The entire area underneath the PR curve can be measured and named average precision (AP) and the mean value of AP of all classes in the model can also be calculated and named mAP (mean AP) (Fig. S2).

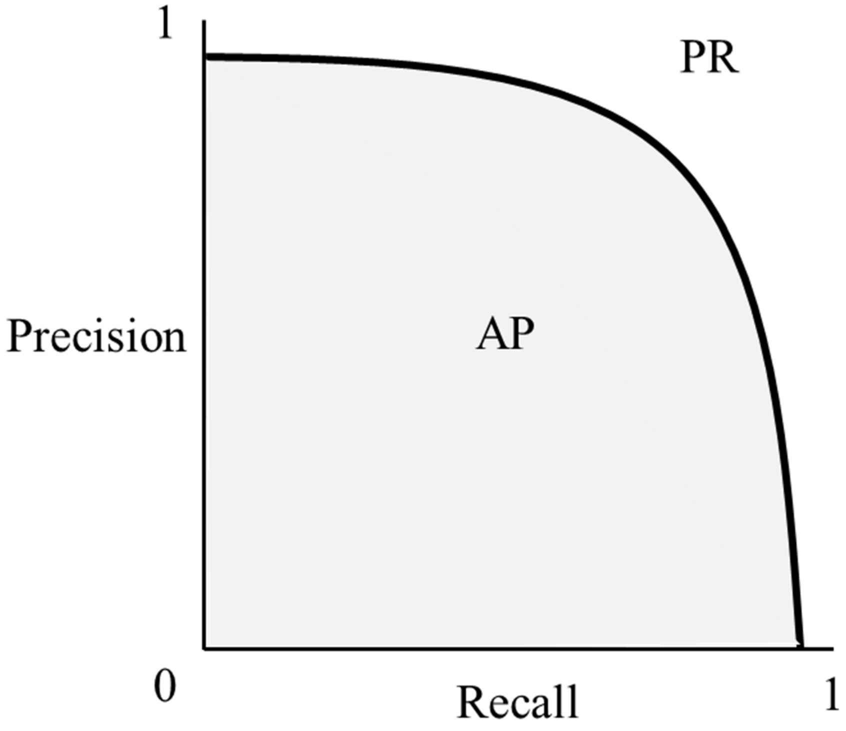

Figure S2.

Precision-recall curve (PR curve) and average precision (AP)

ROC curve and AUC (Area Under the ROC Curve)

The same as PR curve, an ROC curve (receiver operating characteristic curve) is plotted with true positive rate (TPR or recall, y-axis) against the false positive rate (FPR, x-axis, Eq. 4). The entire area under the ROC curve is AUC and the average value of AUC of all classes is macro-average AUC (Fig. S3).

4. $$\text{FPR} = \frac{\text{FP}}{(\text{FP+TN})}$$

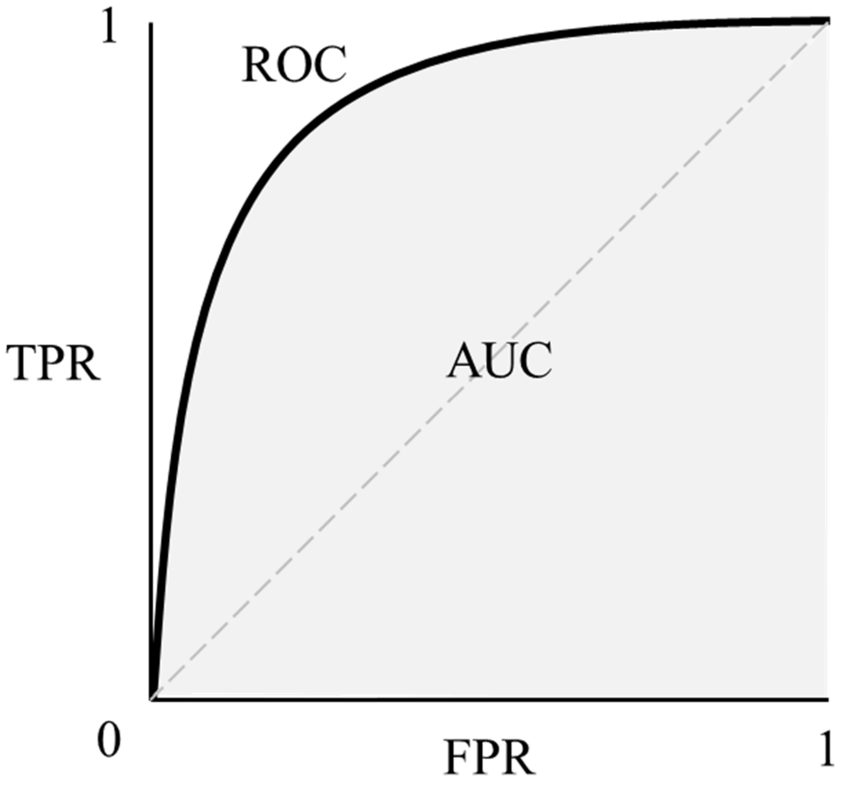

Figure S3.

ROC curve and AUC (Area Under the ROC Curve)
